# Role of Cardiac Biomarkers in Non-Small Cell Lung Cancer Patients

**DOI:** 10.3390/diagnostics13030400

**Published:** 2023-01-22

**Authors:** Valerio Nardone, Alfonso Reginelli, Giuseppina De Marco, Giovanni Natale, Vittorio Patanè, Marco De Chiara, Mauro Buono, Gaetano Maria Russo, Riccardo Monti, Giovanni Balestrucci, Maria Salvarezza, Gaetano Di Guida, Emma D’Ippolito, Angelo Sangiovanni, Roberta Grassi, Ida D’Onofrio, Maria Paola Belfiore, Giovanni Cimmino, Carminia Maria Della Corte, Giovanni Vicidomini, Alfonso Fiorelli, Antonio Gambardella, Floriana Morgillo, Salvatore Cappabianca

**Affiliations:** 1 Department of Precision Medicine, University of Campania “L. Vanvitelli”, 80138 Naples, Italy; 2 Department of Translational Medical Science, University of Campania “L. Vanvitelli”, 80138 Naples, Italy; 3 Radiotherapy Unit, Ospedale del Mare, ASL Napoli 1 Centro, 80138 Naples, Italy

**Keywords:** heart, NSCLC, biomarkers, cardio-oncology, radiotherapy

## Abstract

Treatment-induced cardiac toxicity represents an important issue in non-small cell lung cancer (NSCLC) patients, and no biomarkers are currently available in clinical practice. A novel and easy-to-calculate marker is the quantitative analysis of calcium plaque in the coronary, calculated on CT. It is called the Agatston score (or CAD score). At the same time, other potential predictors include cardiac ultrasonography and anamnesis of the patients. Our work aimed to correlate cardiac biomarkers with overall survival (OS) in NSCLC patients. We retrospectively analyzed patients with NSCLC discussed in the Multidisciplinary Tumor Board of our Institute for the present analysis between January 2018 and July 2022. Inclusion criteria were the availability of basal CT imaging of the thorax, cardiac ultrasonography with the calculation of ejection fraction (EF), and complete anamnesis, including assessment of co-pathologies and pharmacological drugs. The clinical data of the patients were retrospectively collected, and the CAD scores was calculated on a CT scan. All of these parameters were correlated with overall survival (OS) with univariate analysis (Kaplan–Meier analysis) and multivariate analysis (Cox regression analysis). Following the above-mentioned inclusion criteria, 173 patients were included in the present analysis. Of those, 120 patients died in the follow-up period (69.6%), and the median overall survival (OS) was 28 months (mean 47.2 months, 95% CI, 36–57 months). In univariate analysis, several parameters that significantly correlated with lower OS were the stage (*p* < 0.001), the CAD grading (*p* < 0.001), history of ischemic heart disease (*p*: 0.034), use of beta blocker drugs (*p*: 0.036), and cardiac ejection fraction (*p*: 0.005). In multivariate analysis, the only parameters that remained significant were as follows: CAD score (*p*: 0.014, OR 1.56, 95% CI: 1.04–1.83), stage (*p*: 0.016, OR: 1.26, 95% CI: 1.05–1.53), and cardiac ejection fraction (*p*: 0.011, OR 0.46, 95% CI: 0.25–0.84). Both CAD score and ejection fraction are correlated with survival in NSCLC patients at all stages of the disease. Independently from the treatment choice, a cardiological evaluation is mandatory for patients with NSCLC.

## 1. Introduction

Lung cancer is one of the leading causes of cancer mortality worldwide. Only 30% of patients with non-small cell lung cancer (NSCLC) receive an early-stage diagnosis and are eligible for surgical treatment with curative intent; the majority, which represent about 50% of cases, receive the diagnosis in the metastatic stage and are candidates for systemic treatments [1,2,3,4,5]. There are many oncological possibilities in this setting, ranging from chemotherapy to targeted therapy in patients with specific gene mutations, immunotherapy alone or in combination with chemotherapy [6,7,8,9,10,11,12,13]. Patients are eligible for osimertinib (and also gefitinib, erlotinib, afatinib) if they have a classic mutation of the EGFR gene, afatinib (and erlotinib and gefitinib) if they have a mutation of exons 18–21 of EGFR, alectinib (and crizotinib and ceritinib) if there is an ALK rearrangement, crizotinib if there is a rearrangement of ROS1, dabrafenib and trametinib if there is a mutation of BRAF-V600, and entrectinib or larotrectinib in TNRK rearrangement [6,7]. Patients with limited metastases (i.e., oligometastatic patients) should also be treated with systemic and ablative therapies such as radiotherapy [14].

The other 20% of NSCLC patients receive a diagnosis in the third stage of non-small cell lung cancer (NSCLC), which includes a comprehensive and heterogeneous category (IIIA-IIIB-IIIC), in prognostic and therapeutic terms, that can be managed with different strategies in a multidisciplinary approach, such as surgical treatment in selected cases followed by chemotherapy and radiotherapy, neoadjuvant chemotherapy with or without radiotherapy followed by surgical resection, or radical chemoradiotherapy followed by immunotherapy with durvalumab in patients with PD-L1 > 1% [15,16,17,18,19].

The increase in the efficacy of treatments and the survival of patients makes the presence of side effects more important. Treatment-induced cardiac toxicities represent an important issue in thoracic oncology, as they cause mortality and morbidity in cancer survivors [20,21,22,23].

Agatston et al. developed a standardized method, called the Agatston score, for quantification of coronary calcium based on the area of a calcified coronary plaque in a CT slice [24]; the different densities of the plaques are used to stratify the score in four CAD score groups, called CAD grading [25].

Other factors that need to be considered are the presence of co-pathologies, pharmacological therapies, cardiac functionality and other clinical variables.

Our work aimed to retrospectively evaluate the correlation between the prognosis of CAD score and other clinical parameters in lung cancer patients.

## 2. Materials and Methods

Our work was a retrospective analysis of NSCLC patients treated in our unit. The CAD score was calculated on basal CT imaging (i.e., at the time of diagnosis) and was correlated with overall survival together with other clinical variables.

### 2.1. Patient Population

All of the NSCLC patients discussed in the Multidisciplinary Tumor Board (MTB) of thoracic malignancies in our department between January 2018 and July 2022 were included in the present analysis.

- The inclusion criteria were as follows:
- basal pre-treatment CT imaging of the thorax;
- cardiac evaluation, including ultrasonography with the calculation of ejection fraction (EF);
- complete anamnesis, including co-pathologies that could interfere with cardiac functionality;
- pharmacological therapy.

### 2.2. Ethics Approval

All of the patients gave written consent to the anonymous use of their examinations for research scope. The local ethical committee approved the study (Prot. N. 13985/i) as established by national laws. All procedures were undertaken in compliance with the ethical statements of the Helsinki Declaration (2008) of the World Medical Association.

### 2.3. Computed Tomography Imaging

We retrospectively collected and reviewed the first computed tomography (CT) performed at the first diagnosis of NSCLC before performing any therapy (either surgery, radiotherapy or systemic therapy). All CT scans were performed using a 64-detector row CT scanner (Discovery 750 HD, GE Healthcare, Milwaukee, WI, USA). In all patients, CT scans of the chest and abdomen were performed with a spiral technique in the tail–cranial direction (from the bases of the lungs to a plane cutting through a third of the femur, with the patient lying supine). Technical parameters used were: slice thickness, 2.5 mm; beam pitch, 1.375/0.937; reconstruction interval, 0.8 mm; 120–140 kVp and 250–500 mA. A standard reconstruction algorithm was used.

### 2.4. CAC Score Calculation

**Figure 1:**
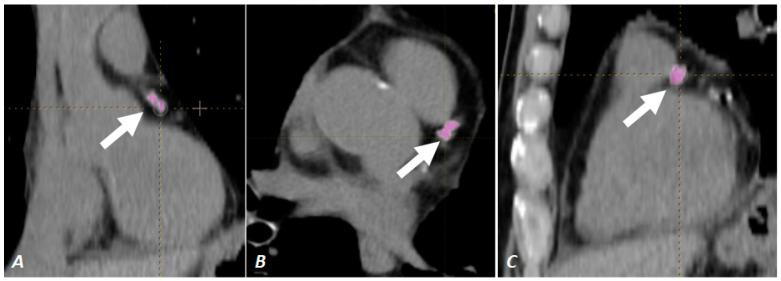
Automatic threshold contouring of coronary calcification on the coronal plane (**A**), axial plane (**B**) and sagittal plane (**C**). The arrow indicates the region of interest of the main coronary artery.

A CAC score was calculated in the region of interest that included the main coronary arteries (i.e., left main, left circumflex, left anterior descending, and right coronary artery) using the Calcium Quantification protocol from LIFEx Software [26] (see Figure 1). Grading of coronary artery disease (CAD grading) was based on a known cut-off of the CAC score (no evidence of CAD: score 0; minimal: score 1–10; mild: score 11–100; moderate: score 101–400; severe: score > 400).

### 2.5. Clinical Variables

The clinical data of the patients were retrospectively collected (age, sex, stage, smoke exposure, co-pathologies: diabetes, dyslipidemia, ischemic heart disease, history of stroke or transient ischemic attack, blood hypertension, pharmacological therapies).

### 2.6. Endpoints and Statistical Analysis

Overall survival (OS) was calculated from each patient’s selected CT scan to the date of death or last follow-up visit. We used the survival analysis (Kaplan–Meier and Cox Rank method) to identify the prognostic parameters related to the outcome endpoints and the log rank to assess the significance of the differences in outcomes, according to the considered SCA score, as well as to clinical parameters. A *p*-value ≤ 0.05 was considered statistically significant. The multivariate analysis was performed with the Cox regression method. A biomedical statistician revised all of the statistical analyses with the SPSS v. 23 software (IBM Corporation, New York, NY, USA) package for Windows.

## 3. Results

Following the above-mentioned inclusion criteria, out of 371 patients discussed in the Thoracic MTB, 173 patients were included in the present analysis; their characteristics are reported in Table 1 and Table 2. One-hundred and twenty patients died at the last follow-up (69.6%), whereas the median OS was 28 months (mean 47.2 months, 95% CI 36–57 months).

### Correlation with Outcomes

In univariate analysis, several parameters significantly correlated with OS: specifically, the CAD grading (*p* < 0.001), the stage of disease (*p* < 0.001), history of ischemic heart disease (*p*: 0.034), use of beta blocker drugs (*p*: 0.036), and cardiac ejection fraction (*p*: 0.005).

**Figure 2:**
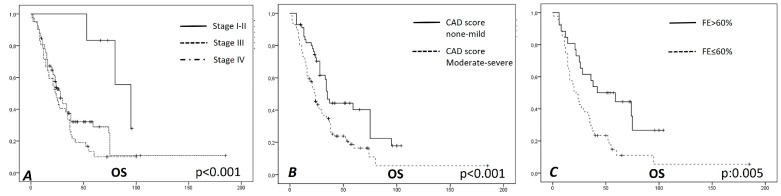
Kaplan–Meier survival analysis for variables significant in multivariate analysis. (**A**) Overall survival (OS) for Stage I–II: median 95 months, mean 84 +/− 6.6 months; OS for Stage III: median 28 months, mean 48 +/− 7.1 months; OS for stage IV: median 23 months, mean 31 +/− 4.2 months, *p*-value < 0.001. (**B**): OS for CAD none–mild: median 35 months, mean 51 +/− 5.2 months; OS for CAD moderate–severe: median 23 months, mean 37 +/− 6.1 months, *p*-value < 0.001. (**C**): OS for cardiac ejection fraction (FE) ≤ 60%: median 22 months, mean 36 +/− 7.1 months; OS for FE > 60%: median 42 months, mean 55 +/− 7.5 months, *p*-value: 0.005.

In multivariate analysis, the only parameters that remained significant were stage (*p*: 0.016, OR: 1.26, 95% CI: 1.05–1.53), CAD score (*p*: 0.014, OR 1.56, 95% CI: 1.04–1.83), cardiac ejection fraction (*p*: 0.011, OR 0.46, 95% CI: 0.25–0.84) (see Table 3 and Figure 2).

## 4. Discussion

Lung cancer has the highest incidence and mortality rate in the world. Recently, the therapeutic scenario for NSCLC not amenable to surgical treatment underwent a radical change determined by immunotherapy in combination with chemoradiotherapy in locally advanced stages or different combinations in metastatic patients [6,7,27,28,29]. Due to the increase in life expectancy, it is paramount to pay more attention to the side effects of therapeutic approaches, such as cardiac toxicity, which appears to be of considerable importance.

Thoracic RT can lead to radiation-induced heart disease (RIHD) in the years following RT [30], and this correlation has been the primary focus of research by numerous studies involving breast cancer and lymphoma patients. In contrast, patients with lung cancer have been less analyzed in this regard due to the shorter life expectancy historically associated with this pathology [30,31]. Notably, locally advanced lung cancer patients treated with chemoradiotherapy and adjuvant immunotherapy also suffer the risk of an increase in immune-related adverse events (irAEs) associated with immunotherapy [32,33]. In this context, several cardiac irAEs have been reported in clinical practice, mainly myocarditis, arrhythmia, pericardial effusion, systemic vasculitis and acute coronary syndrome [32,33].

In our study, the parameters that impact survival in multivariate analysis were the stage of disease, the CAD score and the ejection fraction calculated on cardiac ultrasonography.

The correlation between OS and the stage of disease is quite obvious. Still, it is worth underlining that more patients in the locally advanced or metastatic stages of disease were included in our retrospective analysis.

Several studies have been performed to determine the CAC score and the corresponding CAD risk in patients with different neoplasms [34,35,36,37]. Andersen et al. [35] published the first quantitative study on CAC in long-term survivors of Hodgkin lymphoma (HL) who had survived >15 years after radiotherapy treatment. They related CAC to verified CAD since an increased risk complicates the long-term risk of CAD for HL survivors due to radiation-induced endothelial damage. The first correlation between the CAC score and heart dosimetry in breast cancer survivors treated with radiotherapy was performed by Tjessem et al. [38], who demonstrated that the CAC score is one of the strongest predictive factors for cardiovascular events and is independent of classical cardiological risk factors such as diabetes, hypertension or hypercholesterolemia. These results were then confirmed by subsequent studies [34,39]. Milgrom et al. analyzed the association of CAC score in patients previously irradiated for thoracic malignancies and found that coronary artery dosimetry was strongly correlated with subsequent CAC score, concluding that coronary calcification may occur soon after RT in individuals with conventional cardiac risk factors [20]. In this context, the relationship between RT and CAC score in lung cancer is still under investigation. Miki et al. analyzed the correlation of neoadjuvant chemoradiotherapy (CRT) with the worsening of thoracic aortic calcification using a matched patient approach [40]. Abravan et al. calculated the CAC score on 4D-CT examinations of 334 patients undergoing stereotactic body radiotherapy (SBRT) for early lung cancer [41] and found a statistically significant correlation between calcification volume and overall survival. More recently, Wang et al. retrospectively investigated the cumulative incidence of cardiac events in patients with Stage III NSCLC [42] and found that the volume of calcifications could be used to predict the rates of cardiovascular events. Additionally, in members of the general population that undergo lung cancer screening programs with CT, the CAD score has been shown to be statistically correlated with overall survival [43,44,45].

The ejection fraction is another factor that had an important impact on our study’s outcome. Additionally, Semrau et al. conducted a retrospective analysis of locally advanced NSCLC patients who underwent chemoradiotherapy to investigate the prognostic role of global health status and structural deficits of several organs [46]. The study results show that prognostic factors identified in the multivariate analysis were left ventricular ejection fraction, reduced pulmonary function and T Stage. In addition, many lung cancer patients suffer from concomitant cardiovascular diseases before oncological diagnosis [23], aggravating their prognosis. Similarly, the study by Ren M et al. investigated the influence of atrial cardiomyopathy (ACM) on the prognosis of NSCLC patients and related clinical determinants [47]. It showed that NSCLC patients had a significant ratio of coexisting ACM (17.32%), characterized by a lower ejection fraction and a worse OS. These findings could help us better understand the cardiac burden in these patients and provide additional risk stratification. Another study involved 326 subjects with known cardiovascular disease suffering from lung or breast cancer. In this population, decreased left ventricular ejection fraction was associated with a worse OS [48].

Considering these premises, our study’s results agree with the recent literature. They stress the need for the risk assessment of all NSCLC patients before and during any cancer therapy to improve the results. This knowledge is essential to stratify the risk of developing CVD and start prevention programs and strict cardiologist follow-up in the at-risk population. If the patient develops cardiotoxicity due to treatment for lung cancer, it is necessary to promptly refer the patient to a cardiologist who has experience in treating cardiac complications in thoracic oncology patients.

We recognize as a limit of our investigation the low number of patients analyzed (especially for the early stages of disease) and the study’s retrospective nature. Conversely, the CAC score was calculated on a homogeneous cohort of consecutive lung cancer patients of different stages, using standardized staging with CT imaging of the thorax.

## 5. Conclusions

The prevalence of severe CAC grading is exceedingly high in lung cancer patients amenable to undergoing different treatments. The cardiovascular evaluation for all patients with lung cancer should include different techniques, and patients at risk should be referred to a cardiologist for prevention and strict follow-up of CAD. 

## Figures and Tables

**Table 1:** Clinical characteristics of patients enrolled in the present analysis.

Clinical Parameters	
**Sex** Male Female	
	118 (68.2%) 55 (31.8%)
**Age** 18–50 51–65 66–75 ≥76	
	10 (5.8%) 75 (43.3%) 80 (46.2%) 8 (4.7%)
**Smoke exposure** Unknown Non smoker Previous smoker Actual smoker	
	6 (3.5%) 17 (9.8%) 69 (39.9%) 81 (46.8%)
**Stage of disease** I/II III A III B III C IV	
	7 (4%) 54 (31.2%) 62 (35.8%) 8 (4.6%) 42 (24.4%)

**Table 2:** Cardiological characteristics of the included patients.

Cardiological Characteristics	
**CAD Score** None Mild Moderate Severe	
	40 (23.1%) 17 (9.8%) 18 (10.4%) 98 (56.6%)
**Diabetes** Yes No	
	26 (15%) 147 (85%)
**Dyslipidemia** Yes No	
	38 (21.9%) 135 (78.1%)
**Previous ischemic heart disease** Yes No	
	28 (16.2%) 145 (83.8%)
**TIA or stroke** Yes No	
	8 (4.6%) 165 (95.4%)
**Hypertension** Yes No	
	97 (56%) 76 (44%)
**Sartan use** Yes No	
	34 (19.6%) 139 (80.4%)
**Anticoagulant use** Yes No	
	40 (23.1%) 133 (76.9%)
**Beta blocker use** Yes No	
	36 (20.8%) 137 (79.2%)
**Calcium channel blocker use** Yes No	
	28 (16.2%) 145 (83.8%)
**Diabetes drug use** Yes No	
	16 (9.2%) 157 (90.8%)
**Diuretic use** Yes No	
	26 (15%) 147 (85%)
**Cardiac ejection fraction** ≤60% >60%	
	111 (64%) 62 (36%)

**Table 3:** Multivariate analysis of the significant parameters.

Cox Regression Analysis			
Parameter	*p*-Value	Β	OR (95% CI)
Stage of disease	0.016	0.238	1.26 (1.05–1.53)
CAD score	0.014	0.443	1.56 (1.04–1.83)
Cardiac ejection fraction	0.011	−0.767	0.46 (0.25–0.84)

## Data Availability

The data are available upon request.
